# The Use of EEG in the Study of Emotional States and Visual Word Recognition with or Without Musical Stimulus in University Students with Dyslexia

**DOI:** 10.3390/brainsci16040396

**Published:** 2026-04-06

**Authors:** Pavlos Christodoulides, Dimitrios Peschos, Victoria Zakopoulou

**Affiliations:** 1 Physiology Laboratory, Faculty of Medicine, School of Health Sciences, University of Ioannina, 45110 Ioannina, Greece; pchristo@uoi.gr; 2 Laboratory of New Approaches in Communication Disorders, Department of Speech and Language Therapy, School of Health Sciences, University of Ioannina, 45500 Ioannina, Greece; vzakop@uoi.gr

**Keywords:** dyslexia, EEG, visual recognition, emotional states

## Abstract

This study investigated neural oscillatory dynamics underlying visual word recognition in university students with dyslexia using a portable brain–computer interface (BCI) EEG system. The sample included university students with dyslexia (N = 12) and matched controls (N = 14) who completed auditory discrimination and visual word recognition tasks, with and without musical accompaniment. Through these experimental conditions, the researchers assessed (a) the cortical activation across frequency bands, (b) the modulatory effect of background music, and (c) the relationship between emotional states and brain activity. Results revealed significant group differences in oscillatory patterns, with reduced β- and γ-band activity in the left occipito-temporal cortex among participants with dyslexia, confirming disrupted temporal coordination in posterior reading networks. Compensatory right-hemisphere activation was observed, particularly under musical conditions, accompanied by increased α-band power and reduced δ activity, indicating enhanced attentional engagement and reduced cognitive fatigue. Emotional assessment using the DASS-21 revealed higher stress and anxiety scores in the dyslexic group, suggesting that affective factors may modulate oscillatory dynamics. The presence of background music appeared to attenuate these effects, supporting improved emotional regulation and cognitive focus. These findings demonstrate that dyslexia reflects a distributed disruption in neural synchrony and cross-frequency coupling, influenced by both cognitive and affective mechanisms. The integration of portable EEG technology with rhythmic auditory stimulation offers new insights into the neurophysiological and emotional aspects of dyslexia, highlighting the potential of rhythm- and music-based approaches for both diagnostic and therapeutic applications.

## 1. Introduction

### 1.1. Dyslexia and Visual Word Recognition

Dyslexia is a neurodevelopmental condition that predominantly impairs the development of reading abilities, with a global prevalence estimated at 5–15% among school-aged children and persistent effects extending into adulthood [1,2]. While it has traditionally been defined by deficits in phonological processing, increasing evidence highlights the significant role of visual processing and orthographic recognition impairments in the manifestation of the disorder [3].

Visual word recognition—the process by which the brain deciphers written words—relies on a complex interplay between visual, orthographic, phonological, and semantic systems [4]. In typical readers, the left occipitotemporal cortex, particularly the Visual Word Form Area (VWFA), plays a central role in fast, automatic word recognition [5]. In individuals with dyslexia, activation of the VWFA is often delayed or diminished, accompanied by inefficient recruitment of frontal and temporoparietal regions [6,7]. These anomalies contribute to slower word recognition and increased reliance on effortful compensatory mechanisms.

Galaburda and Livingstone (1993) [8] found that people with dyslexia have smaller and more disorganized magnocellular neurons in the central nervous system compared to typically developing individuals. This was confirmed by Giraldo-Chica et al. (2015) [9], who observed significantly thinner magnocellular layers in the left lateral geniculate nucleus of dyslexic participants. Despite normal vision and optic nerves, these abnormalities slow visual information processing, as documented in neurophysiological and visual-processing studies of dyslexia [10,11]. Stein (2001) [12] also reported dysfunction in the magnocellular region, which is key for perceiving time, visual events, and controlling eye movements. Poor magnocellular function leads to unstable vision and reduced sensitivity to visual motion. As a result, dyslexic individuals often show impaired eye movement control and less accurate eye fixations [13,14].

Despite advances in neuroimaging, dyslexia remains underdiagnosed and under-researched in adult populations, particularly in university settings where compensatory strategies may obscure underlying neurocognitive deficits [15]. Moreover, most existing interventions are designed for children, underscoring the need for targeted research on adult dyslexic readers.

### 1.2. The Use of EEG in Studying Dyslexia

Electroencephalography (EEG) has emerged as a valuable tool in dyslexia research due to its high temporal resolution and non-invasive nature. It captures real-time brain responses to stimuli, allowing researchers to examine millisecond-level neural dynamics during reading tasks [16]. Event-related potentials (ERPs), which are time-locked to specific sensory or cognitive events, offer insight into different stages of processing—ranging from early visual perception (P1, N1) to phonological (N250) and semantic (N400) integration [17,18].

Atypical EEG patterns in dyslexia include altered ERP amplitudes and latencies, often in the left temporoparietal and occipitotemporal regions [19,20]. Resting-state studies have reported increased theta and alpha activity, possibly reflecting inefficient cognitive control and impaired neural synchrony [21]. Furthermore, source localization and coherence analyses reveal disrupted functional connectivity in reading networks [22]. These findings are consistent with recent systematic reviews of EEG studies in dyslexia, which highlight robust alterations in spectral power and hemispheric asymmetries across reading-related tasks [23,24].

Portable EEG systems, such as the Emotiv EPOC+, provide valuable opportunities for conducting ecologically valid and cost-effective research in real-world or semi-structured environments [25]. Recent validation studies have demonstrated that portable EEG systems such as Emotiv can reliably capture group-level oscillatory effects in cognitive tasks, supporting their use in experimental and applied neurocognitive research [26]. Although their spatial resolution is lower than that of clinical-grade systems, their portability and accessibility facilitate data collection in educational and lifestyle contexts, making them particularly well suited for studies involving university students. For example, Eroğlu et al. (2018) [27] employed the Emotiv EPOC+ to develop a neurofeedback-based mobile application aimed at enhancing cognitive functions in children with dyslexia.

Recent work has also explored machine-learning and deep-learning approaches applied to EEG and neuroimaging data for dyslexia screening and classification [28]. These data-driven models can leverage multichannel temporal–spectral representations to detect group-level patterns, often with high classification performance in controlled datasets. However, model performance can be sensitive to dataset size, recording hardware, preprocessing choices, and interpretability constraints, particularly in low-density or portable EEG systems [29,30]. In the present study, we therefore focused on hypothesis-driven spectral power analyses to characterize oscillatory differences during visual word recognition, while acknowledging that future work could combine these physiologically interpretable features with machine-learning pipelines for complementary predictive modeling.

### 1.3. Emotional States, EEG Activity, and Dyslexia

Emotional factors such as depression, anxiety, and stress play a critical role in modulating cognitive and neural processing in individuals with dyslexia. Studies have shown that individuals with dyslexia often experience elevated levels of anxiety and stress, particularly during reading and academic tasks, which can further exacerbate attentional and working memory difficulties [31,32]. Chronic stress and anxiety are associated with altered autonomic regulation and heightened cortical arousal, reflected in EEG patterns of increased beta and high-frequency alpha activity [33]. Conversely, depressive symptomatology has been linked to diminished frontal alpha asymmetry, an indicator of reduced approach motivation and cognitive control [34]. These findings suggest that emotional dysregulation in dyslexia is not merely secondary to learning difficulties but may contribute to broader alterations in cortical oscillatory dynamics.

From a neurophysiological perspective, EEG research has demonstrated that emotional states influence oscillatory activity across multiple frequency bands, modulating attention, inhibition, and executive control [35,36]. In dyslexic populations, atypical theta–alpha coupling and disrupted frontal–parietal connectivity have been observed during tasks requiring cognitive control, suggesting a neural overlap between affective reactivity and reading-related processing [19,21]. Moreover, emotional arousal can either facilitate or hinder task performance depending on the balance between cortical excitation and inhibition—a relationship that may be particularly fragile in dyslexia, where atypical hemispheric specialization and oscillatory instability are well documented [6,10]. Integrating emotional variables into EEG-based studies of dyslexia therefore provides a more comprehensive understanding of how affective and cognitive processes interact to shape neural responses during reading and language-related tasks.

Within Greek populations, a growing body of evidence has highlighted the utility of EEG for identifying functional brain differences in adults with dyslexia. Zakopoulou et al. (2024) [37] reported distinct patterns of functional connectivity during visual word processing, while Christodoulides et al. (2022) [38] demonstrated that oscillatory dynamics in frontal and temporal regions reflect compensatory mechanisms supporting linguistic performance. Similarly, Theodoridou et al. (2021, 2024) [39,40] emphasized the role of emotional regulation and attentional control in mediating cognitive variability among dyslexic adults. Taken together, the above lines of evidence suggest that visual word recognition in dyslexia emerges from the interaction of perceptual, attentional, and affective processes, each of which can be examined through oscillatory EEG dynamics [41]. Background music provides a controlled contextual manipulation that may influence these processes by modulating attentional allocation, arousal, and temporal structure during task performance. Emotional state, in turn, represents an individual-difference factor that may further shape oscillatory responses [42]. The present study integrates these domains to examine how neural oscillations during visual word recognition vary as a function of task context (music vs. no music) and emotional state in adults with dyslexia.

### 1.4. Music and Reading: A Converging Pathway

An emerging literature highlights the overlapping cognitive and neural mechanisms of music and language. Both domains require fine-grained temporal processing, auditory discrimination, rhythm perception, and syntactic structuring [43]. The “OPERA” hypothesis [44] suggests that musical training enhances language processing by inducing neural plasticity in shared auditory and attentional networks.

Rhythmic training appears to benefit phonological awareness—a key deficit in dyslexia—by improving temporal prediction and auditory segmentation [45]. Neurophysiological studies using EEG have shown that music training increases the amplitude and synchrony of auditory ERPs such as the mismatch negativity (MMN) and N1-P2 complex [46]. These enhancements may generalize language tasks, improving phoneme discrimination and orthographic mapping.

Experimental interventions incorporating musical stimuli during reading tasks suggest that background music—especially rhythmic or melodic input—can modulate attention, reduce cognitive load, and enhance reading accuracy, particularly in individuals with learning difficulties [47,48]. However, the neurophysiological mechanisms underlying such effects remain poorly understood, especially in adult dyslexics. Recent experimental and intervention studies have further explored the role of rhythmic training and auditory timing in supporting reading-related processes in individuals with dyslexia, underscoring the relevance of temporal structure in auditory input [49]. In the context of the present study, several commonly used neurophysiological terms are employed in an operational sense. Temporal coordination refers to the timing relationships of neural activity across cortical regions, which are indirectly reflected in frequency-specific oscillatory power rather than directly measured phase synchrony. Neural synchrony is used here to denote coordinated oscillatory activity within specific frequency bands at the group level, as indexed by relative changes in band power across conditions and groups. Cross-frequency coupling is discussed conceptually, referring to interactions between low- and high-frequency oscillations reported in the literature, but was not directly quantified in the present analyses. Finally, attentional engagement is operationalized as task-related modulation of oscillatory power, particularly within alpha, beta, and gamma bands, consistent with prior EEG studies, without implying direct measurement of attentional performance.

### 1.5. The Relationship Between Musical Stimuli and Cognitive Functions

Scientific interest in music’s psychological effects has grown, with most people using music daily for emotional regulation [50,51]. While background music has shown both positive and negative effects on tasks such as reading, memory, and attention [52,53], some studies report improvements in visuospatial and psychomotor performance [54]. Music may enhance memory and emotion through arousal [55], though silence is sometimes more beneficial for cognitive tasks [56]. Meta-analyses have confirmed that music can impair performance in certain contexts [57].

Recent research explores how music affects brain activity and executive functions such as inhibition and attention. Using Go/NoGo tasks, Burkhard et al. (2018) [58] found that background music increased arousal without harming performance. Music can alter brainwaves—techno increased beta activity linked to alertness [59], and Mozart improved task performance via prefrontal cortex activation [60]. Still, gaps remain in understanding how music genre, cognition, and physiology interact, especially in populations like individuals with dyslexia, who have been largely overlooked in studies involving reading and music.

The use of background music in cognitive tasks has been motivated by evidence that structured auditory input can influence attentional allocation, arousal, and temporal processing. In the present study, a single musical piece was selected to provide a controlled and consistent auditory context across participants, rather than to induce training or learning effects. Mozart’s Sonata for Two Pianos in D major, K.448, was chosen due to its regular tempo, clear rhythmic structure, and frequent use in prior EEG and cognitive studies examining auditory context effects. Importantly, the present study does not assume a specific “Mozart effect,” nor does it equate passive listening with rhythmic training or active musical engagement. Instead, background music is treated as a contextual auditory factor that may modulate ongoing neural activity during task performance.

These findings can be interpreted within the framework of the Temporal Sampling Theory [45], which posits that dyslexia arises from impaired phase-locking of low-frequency neural oscillations (delta, theta) to speech rhythms. Such temporal sampling deficits are thought to hinder the extraction of phonological cues, thereby impairing grapheme–phoneme correspondence [61,62]. Evidence further suggests that musical stimuli, particularly rhythmic structures. may entrain neural oscillations, enhance temporal prediction, and facilitate phonological processing [63,64,65]. This rhythmic entrainment is proposed to reduce cognitive load during reading by improving auditory–motor synchronization and strengthening the neural encoding of speech.

Furthermore, the observed right-hemispheric activations align with neurocompensatory models, which suggest that adults with dyslexia may recruit non-dominant regions to support deficient left-lateralized language networks [66,67]. The increased reliance on visual and attentional resources in the right hemisphere, particularly under the modulation of music, supports the hypothesis of cross-modal neural plasticity in response to environmental scaffolding.

### 1.6. Significance and Novelty of the Study

To our knowledge, this is one of the first studies to systematically examine the effects of musical stimuli on visual word recognition in university students with dyslexia, using portable EEG technology. The combination of a psycholinguistic discrimination task with simultaneous musical input offers a novel paradigm for exploring auditory-visual interactions in reading.

Moreover, the use of spectral power analyses (alpha, beta, gamma bands) provides a nuanced understanding of how music modulates attention, memory, and perceptual processing during reading. The study also addresses a significant gap in the literature by focusing on adult learners, a population often overlooked in dyslexia research.

These findings have implications for the development of multisensory reading interventions, educational accommodations, and neurofeedback-based training protocols tailored to adult learners with reading difficulties.

### 1.7. Objectives

Accordingly, the present study aimed to investigate how neural oscillatory activity during visual word recognition differs between university students with and without dyslexia, and how this activity is associated with (a) the presence of background music as a contextual auditory factor and (b) individual differences in emotional state. Using portable EEG, the study focuses on frequency-band–specific oscillatory patterns as indices of attentional, perceptual, and affective processing, without implying causal or intervention effects. Specifically, the study sought to address the following research questions:

• Are cortical activation patterns different across frequency bands (delta, theta, alpha, beta, and gamma) between dyslexic and control participants during visual recognition?
• Does the presence of background music modulate neural oscillations during visual word recognition?
• Is there a relationship between emotional states (depression, anxiety, and stress, as measured by DASS-21) and oscillatory brain activity during visual word recognition with and without background music?

## 2. Materials and Methods

### 2.1. Participants

The sampling methodology used in this study is stratified random sampling. A total of 26 right-handed university students (mean age 21.32 y/o) participated in this experiment, forming the Dyslexic group (12 students) and the Control group (14 students). Participants were recruited from the undergraduate student population of the University of Ioannina through course announcements and student support services. The study did not involve screening of the entire student population; rather, participants self-selected based on eligibility criteria and availability. Group assignment (dyslexic vs. control) was based on documented diagnostic history rather than random stratification. The diagnosis of dyslexia was based on prior formal assessment documented in participants’ educational records. No additional standardized diagnostic batteries were administered as part of the present study. The absence of comorbid conditions was determined through self-report and participant history, rather than systematic clinical screening, and should therefore be interpreted cautiously. All participants were native speakers of Greek. A history was taken from all participants where general information such as educational level, occupational status and their native language was recorded, as well as more specific information about their educational background, i.e., whether they attended an inclusion class or received speech and language therapy intervention. Information was also provided on any behavioral problems during school age through a brief psychosocial history. At the end of the procedure, each participant received a certificate of participation.

All the subjects with dyslexia had undergone intervention at a young age without reporting any dyslexia-related comorbidities. References to intervention pertain to prior educational or therapeutic support received during childhood or adolescence and do not refer to any intervention administered as part of the present study. There were no major age or education-level differences since all of them were university students in the School of Health Sciences. The study was approved by the Ethics Committee of the University of Ioannina, and all participants provided written informed consent prior to participation.

### 2.2. Experimental Design and Stimuli

For the purposes of the research, a software was created which was used for the electronic presentation of visual stimuli in the phonological test. Stimulus presentation and task execution were implemented using custom software developed in Python 3.8, incorporating standard experimental control libraries. The software monitored the participants’ responses on two experimental conditions: (a) visual discrimination, and (b) visual discrimination with the accompaniment of background music. In the first condition (namely, the visual discrimination), the participants saw different words on the screen and were instructed to read them as carefully as possible and choose the one that seemed right to them. Again, the non-words contained mistakes concerning the position and the order of the letters, like sequential (fridge–frigde), insertion (computer–compluter), omission (bicycle–bicyle), or letter substitution errors (dog–tog). The second experimental condition was like the previous one, with the difference being the simultaneous presence of musical accompaniment. The musical excerpt selected for the experiment was the Sonata for Two Pianos in D major, K. 448, a work composed by Mozart. Participants were not instructed to attend to or synchronize with the music; the musical stimulus was presented as a passive background auditory context during task performance. The musical stimulus was delivered via headphones at a fixed, comfortable volume level across participants, which was set prior to task onset and not adjusted during the experiment. Volume levels were set to a comfortable listening level prior to task onset and kept constant across participants to reduce variability in auditory stimulation.

The material presented in the two conditions followed several predefined phonological and morphological criteria based on commonly made mistakes in the Greek language by individuals with dyslexia, especially focusing on confusion of letters with visual/spatial (λ, γ, χ) or phonemic similarity (f, v, θ, ð). Word stimuli were selected from commonly used Greek lexical items appropriate for adult readers. Pseudowords were constructed by the authors based on phonological and orthographic patterns typical of Greek dyslexic errors (e.g., letter substitutions, transpositions, and omissions), ensuring they were pronounceable but non-lexical. Stimuli were selected to minimize gross differences in word length and visual complexity between real words and pseudowords [68,69]. However, fine-grained psycholinguistic properties such as lexical frequency and bigram frequency were not systematically matched, and this constitutes a limitation of the stimulus design. Specifically, in each condition, the participants had to choose the correct word among a group of three words (1 real Greek word and 2 pseudowords). The average time for each response was 8.5 s.

The whole experimental session lasted 22 min on average, depending on the time required by the participants to answer each question. The recording was terminated as soon as a participant felt any discomfort with the device or the procedure. Although rest breaks were provided between conditions, the order of task blocks was not counterbalanced. As a result, potential effects of task repetition, habituation, or fatigue cannot be fully excluded and are considered in the interpretation of the results.

**Figure 1:**
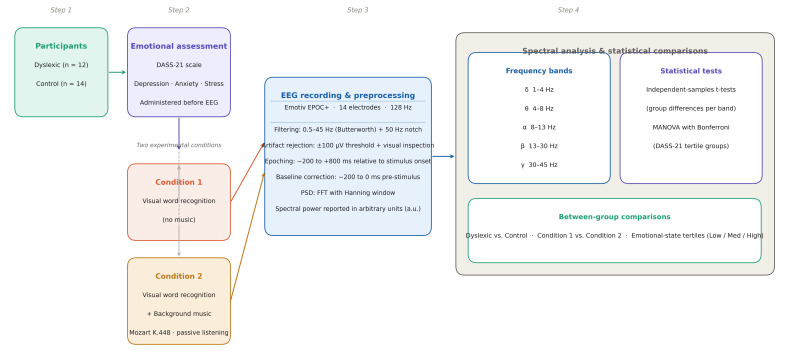
Study workflow illustrating participant recruitment, emotional assessment (DASS-21), experimental conditions, EEG acquisition and preprocessing, and spectral power analysis.

A schematic overview of recruitment, task conditions, EEG acquisition, and analysis workflow is provided in the following study workflow (Figure 1).

### 2.3. DASS-21 Scale

Participants in both groups (Control and Dyslexic) were evaluated using the Depression, Anxiety, and Stress Scale–21 Items [70]. This self-report instrument assesses three major emotional dimensions: depression, reflecting dysphoria, hopelessness, devaluation of life, self-depreciation, lack of interest, anhedonia, and inertia; anxiety, encompassing autonomic arousal, musculoskeletal tension, situational anxiety, and subjective feelings of anxious affect; and stress, which captures chronic, non-specific arousal and irritability. The DASS-21 was developed not merely to replicate conventional measures of emotional distress, but to refine the conceptual and empirical distinction between depression, anxiety, and stress, and to advance their precise quantification in both research and clinical contexts. The scale has been standardized and validated for the Greek population, demonstrating excellent internal consistency reliability (Cronbach’s α = 0.965) [71]. The DASS-21 questionnaire was administered prior to the EEG experiment.

### 2.4. EEG Acquisition

**Figure 2:**
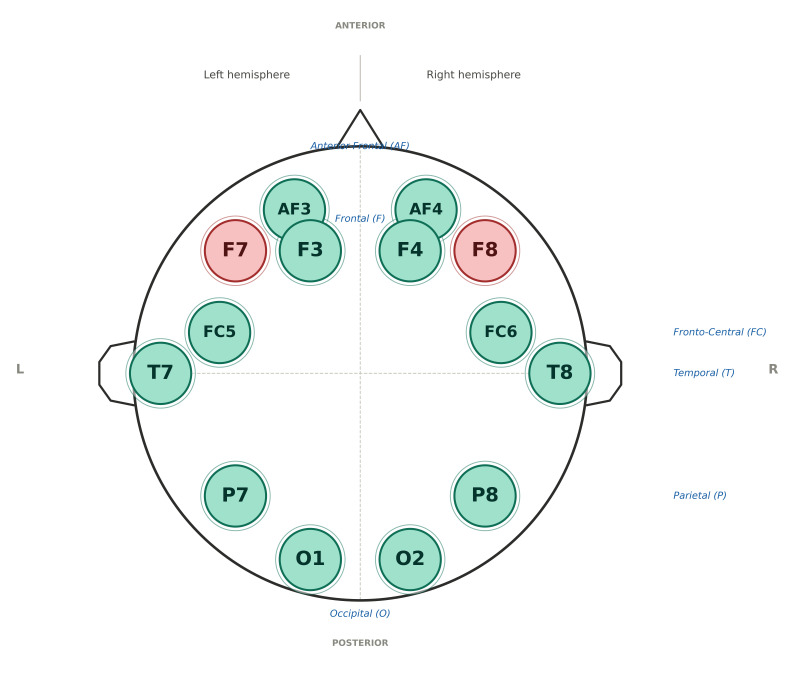
Electrode placement of the Emotiv EPOC+ EEG headset according to the International 10–20 System. Odd numbers denote the left hemisphere; even numbers denote the right hemisphere. Electrodes F7 and F8 (shown in red) were excluded from analysis due to persistent loss of connectivity; the remaining 12 electrodes (shown in green) were retained.

For the EEG recordings, the BCI device Emotiv EPOC+ headset was employed, a wireless neuro-signal acquisition system equipped with 14 wet sensors, which has been validated for research applications in cognitive neuroscience and brain–computer interface studies [72,73,74,75], capable of detecting brainwaves at a 128 Hz sequential sampling rate. The participants were seated in a comfortable chair in front of a computer screen, and a specialized technician set up the device following the instructions provided by the EmotivPRO Software (EMOTIV EPOC+), regularly checking the quality of the connectivity in the beginning and during the recording. The felt pads were placed on the scalp according to the International 10–20 System (AF3, F3, F7, FC5, T7, P7, O1, AF4, F4, F8, FC6, T8, P8 and O2), using a saline liquid solution on all felt pads of each sensor (Figure 2). However, due to loss of connectivity, the F8 electrode was isolated and rejected, and so was the corresponding channel, F7, to maintain the symmetry of the recording. Signal quality was continuously monitored throughout the recording using the EmotivPRO software. Electrode contact was checked prior to and during the session, and segments with poor signal quality or excessive noise were excluded during preprocessing. Rest breaks were provided between task blocks to reduce fatigue. Raw EEG signals were bandpass filtered using a second-order Butterworth filter with a passband of 0.5–45 Hz to remove slow cortical drift and high-frequency noise. A notch filter was additionally applied at 50 Hz to suppress powerline interference. Artifact rejection was performed in two stages: first, epochs containing peak-to-peak amplitudes exceeding ±100 µV were automatically excluded; second, remaining epochs were visually inspected and any segments showing residual ocular, muscular, or movement-related artifacts were rejected manually. Continuous EEG data were segmented into epochs time-locked to the onset of each visual stimulus, spanning −200 ms to +800 ms relative to stimulus onset. A pre-stimulus baseline period of −200 ms to 0 ms was applied for baseline correction, whereby the mean baseline amplitude was subtracted from each epoch. Power spectral density (PSD) was estimated for each epoch using a Fast Fourier Transform (FFT) with a Hanning window to minimise spectral leakage. Mean spectral power was then averaged across accepted epochs for each participant, condition, electrode, and frequency band (δ: 1–4 Hz; θ: 4–8 Hz; α: 8–13 Hz; β: 13–30 Hz; γ: 30–45 Hz). Spectral power values are reported in arbitrary units (a.u.), as is common for power spectral density estimates derived from portable EEG systems. All preprocessing and spectral analyses were performed uniformly across participants and conditions to ensure comparability of oscillatory measures. No separate resting-state EEG recording was acquired; consequently, analyses focus on task-related oscillatory activity and do not allow direct comparison with baseline neural states.

### 2.5. Statistical Analysis

Given the exploratory nature of the study and the limited sample size, no priori power analysis was conducted. The study was therefore not designed to detect small effect sizes, and null findings should be interpreted with caution. Effect sizes are reported throughout to support interpretation of the observed group differences.

Given the number of frequency bands and regional comparisons examined, results were interpreted with caution. To mitigate the risk of inflated Type I error, statistical significance was evaluated in conjunction with effect size estimates and consistency of patterns across conditions, rather than relying solely on uncorrected *p*-values. This approach is consistent with exploratory EEG research employing hypothesis-driven frequency-band analyses.

DASS-21 scores were categorized into low, medium, and high groups using tertile splits to enable exploratory comparisons across emotional-state levels. This approach was chosen to facilitate group-level analysis given the limited sample size and is not intended to imply clinical categorization. For each DASS-21 subscale, scores were divided into tertiles based on the distribution of the full sample, yielding low, medium, and high categories. Grouping was not performed separately for dyslexic and control participants, as the analysis aimed to examine emotional-state effects across the combined sample rather than apply clinical cut-off criteria.

For independent-samples *t*-tests, degrees of freedom reflect the total sample size minus one (df = N − 1 = 25), as reported by the statistical software.

## 3. Results

Prior to analysis, data were tested and confirmed to be approximately normally distributed. Independent *t*-tests were therefore applied to compare group means for each brain region and frequency band. Multiple comparisons were not performed, as each test addressed a distinct brain area and oscillatory rhythm. All statistical analyses reported below assess between-group differences or group-related effects. All reported effects reflect differences in mean oscillatory power between groups or conditions and should not be interpreted as correlational relationships.

To compare cortical activation patterns across frequency bands (delta, theta, alpha, beta, and gamma) between dyslexic and control participants during visual recognition, we examined the relationship between brain regions and oscillatory frequency bands under two experimental conditions, such as: (i) visual word recognition and (ii) visual word recognition with background music. It should be noted that independent-samples *t*-tests were conducted for all cortical regions and frequency bands in both hemispheres under each experimental condition. Table 1 and Table 2 report only those comparisons that reached statistical significance (*p* < 0.05); regions and bands for which no significant between-group difference was found are not displayed, but were nonetheless included in the analysis. In the visual-only condition, statistically significant group differences were identified across several cortical regions. In the left hemisphere, significant effects emerged in the temporal lobe for beta (β) and gamma (γ) bands, in the occipital lobe for alpha (α), beta (β), and delta (δ) bands, and in the parietal lobe for the beta (β) band. In the right hemisphere, significant differences were detected in the occipital lobe for delta (δ), beta (β), and gamma (γ) rhythms. Participants with dyslexia exhibited higher mean power values only in the delta band of both left and right occipital regions (see Table 1). Compared to controls, participants with dyslexia exhibited increased delta power and reduced beta and gamma power in the specified regions.

Under the conditions incorporating background musical stimulation, significant differences were also observed between groups, though these were largely restricted to the right hemisphere. Specifically, the prefrontal region (AF4) showed group differences in the beta (β) band, the frontal region (F4) in alpha (α), the frontal-central region (FC) in alpha (α), the temporal region in alpha (α), and the occipital region in gamma (γ) (see Table 2).

**Figure 3:**
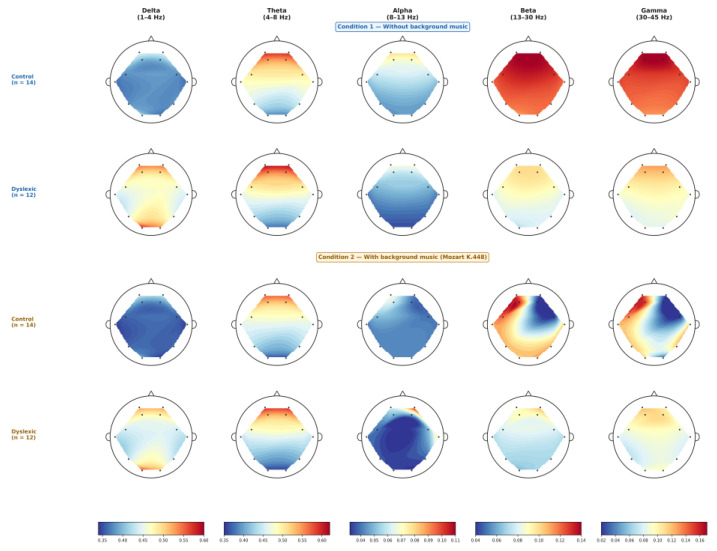
Scalp-level topographic maps of mean spectral power (a.u.) across five frequency bands (Delta, Theta, Alpha, Beta, Gamma) for the Control (n = 14) and Dyslexic (n = 12) groups under Condition 1 (visual word recognition without background music) and Condition 2 (visual word recognition with background music). Colour scales are band-specific (blue = lower power; red = higher power). Electrode positions reflect the 12 active sensors of the Emotiv EPOC+ system.

To facilitate interpretation of the tabulated results, scalp-level topographic visualizations of oscillatory power are provided in Figure 3. These maps illustrate the spatial distribution of group-averaged spectral power (and/or between-group differences) across the principal frequency bands implicated by the statistical comparisons.

Emotional-state effects were examined using multivariate analysis of variance (MANOVA) with tertile-based grouping of DASS-21 scores (low, medium, high). The MANOVA results are presented across four tables, organised by experimental condition and emotional subscale. Table 3 and Table 4 report findings from the visual-only condition (visual word recognition without background music), with Table 3 addressing the Anxiety subscale and Table 4 addressing the Stress subscale. Table 5 and Table 6 report the corresponding findings from the music condition (visual word recognition with background music), with Table 5 addressing Anxiety and Table 6 addressing Stress. Depression did not yield statistically significant effects under either condition and is therefore not presented in tabular form. This structure parallels the organisation of the between-group *t*-test results in Table 1 and Table 2, and allows direct comparison of emotional-state effects across the two experimental conditions. Post hoc comparisons were conducted using the Bonferroni correction to identify significant within-factor differences and control for Type I error in the two experimental conditions:

- During the visual-only condition, several significant differences were observed between the Control and Dyslexic groups, primarily related to Anxiety and Stress, whereas Depression yielded no significant effects. Under the Anxiety condition, dyslexic participants demonstrated higher mean power in the delta (δ) and alpha (α) bands across the frontal, parietal, occipital, and temporal regions of the left hemisphere (*p* < 0.05), as well as in the right parietal and occipital regions. Additional between-group differences were identified in the gamma (γ) band within the occipital cortex, reflecting distinct high-frequency activation patterns between groups (see Table 3). Under the Stress condition, significant effects were found primarily in the right hemisphere, where dyslexic participants exhibited increased activity in the frontal δ and α bands and parietal δ and α bands (*p* < 0.05) (see Table 4).

ii. In the visual recognition task performed with background music, significant group differences between the Control and Dyslexic participants were primarily observed in relation to Anxiety and Stress, while Depression again showed no significant effects. Under the Anxiety condition, dyslexic individuals demonstrated higher mean power values in the left frontal δ (delta) and left frontal α (alpha) bands compared with controls, as well as increased activity in the left occipital δ (delta) and left occipital α (alpha) bands (see Table 5). Conversely, the left temporal α (alpha) band showed significantly greater activation in the Control group. In the right hemisphere, the dyslexic group exhibited elevated occipital δ (delta) power, while right frontal δ (delta) activity differed significantly under the Stress condition. These findings indicate that, even in the presence of background music, group-related differences in neural oscillations persisted, particularly within the delta and alpha frequency bands of the frontal and occipital cortices (see Table 6).

## 4. Discussion

### 4.1. Cortical Activation Patterns

The primary aim of this study was to examine neural oscillatory activity across regions of interest by comparing EEG-recorded rhythms between university students with dyslexia and age-matched controls under two experimental conditions: (i) visual word recognition and (ii) visual recognition accompanied by background music. The current results extend prior Greek EEG studies of dyslexia [37,38] by demonstrating that both visual and affective processing contribute to atypical oscillatory dynamics in adults. While earlier work primarily emphasized phonological and visual pathways, our findings reveal how emotional states such as depression, anxiety, and stress interact with neural rhythms across cortical regions. The present study did not include behavioral performance indices such as reaction times or accuracy measures during the visual word recognition task. As a result, interpretations linking oscillatory activity to attentional engagement, fatigue, or phonological efficiency are necessarily indirect and based on established associations in the EEG literature rather than task-specific behavioral validation. Consequently, the reported neural patterns should be interpreted as reflecting differences in oscillatory dynamics across groups and conditions, without assuming corresponding differences in overt performance. Throughout the Discussion, references to attentional engagement, neural synchrony, or temporal coordination are therefore intended as descriptive interpretations of frequency-band–specific oscillatory patterns, rather than direct measures of cognitive or neural coupling processes.

In general, the present findings demonstrate that consumer-grade EEG devices can detect meaningful group-level differences in brain activity between typically developing adults and those with dyslexia, particularly in frequency bands linked to visual recognition. Importantly, the results across both experimental conditions converge with prior neuroimaging evidence of left occipito-temporal hypoactivation, a hallmark neural signature consistently associated with reading difficulties and phonological decoding deficits in dyslexia [76]. Collectively, these outcomes support the feasibility of using wearable EEG technology to capture the neurophysiological correlations of dyslexia in real-world and multimodal task contexts.

In response to the first research question, the results revealed significant bilateral hemispheric differences, with reduced β- and γ-band activity in the left occipito-temporal cortex among participants with dyslexia. These findings are consistent with previous neuroimaging and electrophysiological studies showing hypoactivation of left posterior cortical regions, particularly within the reading network encompassing the occipito-temporal and temporo-parietal areas [66,76]. Such reduced activation is associated with impaired phonological decoding and orthographic integration, reflecting a disruption in the neural specialization required for fluent reading. It is important to note that changes in delta, beta, and gamma activity likely reflect different aspects of task-related neural dynamics rather than a single underlying process. Delta-band activity may be sensitive to broader cognitive state, task engagement, or sustained processing demands, whereas beta and gamma activity have been more frequently linked to higher-frequency coordination and language-related processing. In the present study, these distinctions are used descriptively to characterize patterns of oscillatory variation rather than to imply distinct functional mechanisms. Although delta-band activity is often associated with drowsiness or low arousal, converging evidence indicates that delta oscillations are also involved in task engagement, sustained attention, and effortful cognitive processing, particularly during demanding or controlled tasks [77,78,79]. In the context of the present study, increased delta power in dyslexic participants may reflect either greater effortful processing demands during the visual word recognition task or a more persistent state of reduced cortical arousal. In the absence of a resting-state baseline recording, these two accounts cannot be formally distinguished. Accordingly, this finding is interpreted cautiously as indicating altered oscillatory dynamics in dyslexic participants, without attributing it definitively to either mechanism.

Conversely, compensatory β- and γ-band hyperactivation was observed in the right hemisphere, particularly in frontal and parietal regions, supporting the notion of functional reorganization in adults with dyslexia. Similar right-hemispheric engagement has been reported in recent ERP studies conducted in transparent orthographies [80], indicating reliance on alternative processing routes for grapheme–phoneme conversion. These compensatory mechanisms likely reflect adaptive neural plasticity that persists in adulthood. Comparable asymmetrical activation has also been described in patients with acquired phonological dyslexia following brain injury, where compensatory recruitment of contralateral regions supports phonological decoding [37].

At the oscillatory level, atypical activation was evident across multiple frequency bands. Reduced β and γ rhythms may indicate impaired temporal coordination of neuronal populations involved in phonological encoding, consistent with the temporal sampling framework [45,81]. Prior EEG research has reported abnormal phase-locking and reduced cross-frequency coupling between delta, theta, and higher-frequency bands in dyslexic readers, which has been linked to difficulties in extracting speech-relevant cues [82]. Although cross-frequency coupling was not directly measured in the present study, the observed spectral power differences are consistent with this literature. Consistent with the findings of Keshavarzi et al. (2025) [83] and Boemio et al. (2005) [84], the elevated mean power observed in the delta band over both left and right occipital regions suggests atypical oscillatory dynamics in response to larger phonological units. This pattern further supports the proposed involvement of delta-band activity in the temporal parsing and rhythmic structuring of speech at the phrasal level [85]. Further evidence from Christodoulides et al. (2022) [38] confirmed that EEG classification using a BCI device and linguistic software can reliably discriminate young adults with dyslexia, reinforcing the potential of portable EEG for adult assessment. Although prior studies have associated alpha, beta, and gamma band activity with attentional and language-related processes, and delta band activity with broader task engagement or cognitive state, such associations are context-dependent. In the absence of concurrent behavioral measures, these interpretations should be considered descriptive and hypothesis-generating rather than confirmatory. In the present study, the observed oscillatory deficits in left posterior regions support this interpretation, suggesting that disruptions in temporal synchronization extend beyond auditory processing into visual word recognition. Throughout the following discussion, interpretations of oscillatory activity are framed as associations derived from group-level EEG patterns and should not be taken to imply causal relationships, performance enhancement, or intervention efficacy.

The heterogeneity of oscillatory responses across α, β, δ, and γ bands reinforces the multifactorial nature of dyslexia [86]. Rather than a uniform deficit, dyslexia appears to involve distributed disturbances in cortical timing and connectivity across visual, phonological, and attentional systems. This aligns with recent computational evidence showing that explainable deep-learning models trained on EEG data can identify dyslexia-specific synchronization patterns with high accuracy [82]. Collectively, these findings indicate that dyslexia is associated with both localized reductions in oscillatory power in left posterior systems and broader alterations in frequency-band activity across hemispheres. These patterns are consistent with prior proposals of disrupted cross-frequency neural coordination in dyslexia, although such coupling was not directly measured in the present study.

### 4.2. Background Music and Neural Modulation

Regarding the second research question, the results demonstrated increased α-band activation and decreased δ activity in the right hemisphere under musical conditions, particularly in frontal, temporal, and parietal areas. This pattern suggests enhanced attentional engagement and cognitive control facilitated by the rhythmic properties of the Mozart K.448 stimulus.

These findings align with earlier research by Lin et al. (2014) [87], who observed significant increases in cortical arousal during music listening, particularly reflected in α-band modulation. The α rhythm is often interpreted as a marker of attentional regulation and inhibitory control [21]; its enhancement under musical conditions indicates that participants with dyslexia may rely on music-induced attentional stabilization to manage the cognitive demands of reading tasks. The reduction in δ power in parietal regions further supports the idea of increased alertness and reduced cognitive fatigue during music exposure, consistent with the association between δ suppression and heightened vigilance [88].

The results also resonate with behavioral findings from Descamps et al. (2025) [49], who demonstrated that rhythm training significantly improved reading accuracy and speed in children with dyslexia. Similarly, Flaugnacco et al. (2015) [65] found that rhythmic perception and production predicted reading ability, suggesting shared neural mechanisms between musical rhythm and phonological processing. The modulation of oscillatory activity observed in the present study is consistent with prior proposals that rhythmic auditory input can entrain neural oscillations and enhance temporal prediction [89,90], although entrainment per se was not measured in the present analyses. The observed spectral power differences between musical and non-musi8cal conditions are therefore interpreted as suggestive of modulation effects rather than as direct evidence of entrainment. This mechanism supports the hypothesis that music acts as an external temporal scaffold, facilitating cross-modal integration between auditory and visual networks [91,92].

Moreover, the finding that right-hemisphere activation persisted under musical stimulation indicates that music may engage compensatory systems supporting visual attention and perceptual integration. Evidence from cross-modal neuroimaging studies confirms that auditory cues, including speech and music, can prime visual representations and facilitate semantic access [93]. The musical background in this study may have enhanced right-hemispheric attentional networks, enabling dyslexic participants to allocate cognitive resources more efficiently during the visual recognition task. This interpretation aligns with recent rhythm-based studies in dyslexia, although the present findings should be considered exploratory [49].

These results collectively highlight the potential of rhythm and music-based interventions as neuromodulatory tools for enhancing attentional control and reading performance. By synchronizing cortical oscillations and improving temporal precision, music may compensate for deficits in phonological and visual processing. This interpretation aligns with theories of neural entrainment that emphasize the capacity of rhythmic stimuli to promote large-scale brain synchronization [63,94]. The integration of musical stimulation in EEG paradigms thus provides valuable insights into how external rhythms can dynamically modulate neural activity and cognitive performance in dyslexia. Accordingly, references to attentional engagement, fatigue, or processing efficiency throughout the Discussion are intended as interpretive frameworks grounded in prior literature, rather than direct inferences from the present data.

### 4.3. Emotional States and Oscillatory Activity

Answering the third research question, it was found that participants with dyslexia exhibited higher mean scores for anxiety and stress compared to controls, reflecting elevated emotional reactivity and attentional dysregulation often associated with reading difficulties [40]. This emotional profile may contribute to variability in oscillatory activation, as anxiety and stress are known to modulate cortical rhythms involved in attention and executive control.

Neurophysiological studies have demonstrated that emotional arousal affects α and β activity, influencing cognitive performance and attentional stability [21]. In this context, the increased α-band activation under musical conditions observed in dyslexic participants may not only reflect cognitive engagement but also reduced emotional arousal facilitated by the calming and predictable structure of Mozart’s composition. Music has long been shown to regulate affective states, decreasing stress and anxiety while enhancing mood and motivation through its modulation of limbic and reward circuits [94].

The interaction between emotional regulation and oscillatory activity observed in this study suggests that music may exert dual benefits: stabilizing both attentional and affective neural systems. The right-hemisphere α synchronization under musical stimulation could reflect improved emotional self-regulation, reducing interference from anxiety-related distractibility. Furthermore, β and γ hypoactivity in left posterior regions—linked to reduced phonological efficiency—may also be partially influenced by emotional strain, as stress can suppress task-related oscillatory coherence. By promoting relaxation and attentional focus, background music may mitigate such effects, enabling more efficient recruitment of compensatory networks.

These findings underscore the importance of considering affective factors in dyslexia research. Emotional states not only modulate cortical activation but also influence the efficacy of cognitive interventions. Integrating music-based strategies that simultaneously target emotional regulation, and temporal synchronization could thus optimize learning outcomes and enhance neuroplasticity in individuals with dyslexia.

### 4.4. Strengths and Limitations

A major strength of the present study lies in its innovative experimental design, which integrated auditory and visual processing tasks with simultaneous EEG recordings to investigate oscillatory dynamics in adults with dyslexia. By incorporating musical stimuli into the paradigm, the study extends current research on the neural mechanisms of dyslexia and provides novel insights into the potential influence of rhythmic auditory input on cortical modulation. The application of multivariate analytical methods further strengthened the reliability of the results by capturing complex, region-specific patterns of neural activation across hemispheres.

Despite these strengths, several limitations should be acknowledged. The modest sample size (12 adults with dyslexia and 14 controls) limits the generalizability of the findings, although comparable cohort sizes are common in exploratory neurocognitive and educational research [95]. In addition, the absence of a resting-state EEG condition constrained direct baseline comparisons, making it difficult to determine whether some observed group differences reflect task-specific modulation or more general neural traits. In particular, the elevated delta power observed in dyslexic participants cannot be unambiguously attributed to task-driven effortful processing versus a trait-like state of reduced vigilance; future studies should include a resting-state baseline recording to enable this distinction. Although rest breaks were provided, task repetition and fatigue effects cannot be fully excluded and may have contributed to variability in signal stability. In addition, behavioral indices of task performance, such as accuracy rates or reaction times, were not recorded in the present study, which limits the ability to directly relate oscillatory activity to reading performance. The limited spatial coverage of the Emotiv EPOC+ system, including the absence of central midline electrodes, restricts the ability to examine midline components associated with attention and error monitoring. Accordingly, interpretations are limited to regional oscillatory patterns rather than fine-grained source-level or ERP-based inferences. Furthermore, the present analyses were restricted to band-specific spectral power and did not include direct measures of phase synchrony, cross-frequency coupling, or functional connectivity. Interpretations referencing these constructs throughout the manuscript are therefore conceptual, grounded in the broader EEG literature, and should be tested directly in future work using phase-based and connectivity analyses.

An additional limitation concerns individual differences in music perception and response. Factors such as musical preference, familiarity with the selected piece, emotional valence, and habitual listening practices were not assessed or controlled for, and may have influenced neural responses to background music. Consequently, the observed associations between music and oscillatory activity should be interpreted at the group level and may not generalize uniformly across individuals. Future studies should replicate these findings in larger and more heterogeneous samples, incorporate resting-state and longitudinal designs, and explicitly measure or manipulate musical parameters to disentangle their contribution to task-related neural dynamics over time.

These findings primarily inform theoretical understanding of neural variability during visual word recognition and highlight directions for future research, rather than supporting immediate applied or clinical implementation.

### 4.5. Practical Implications

The observed associations between background music and oscillatory activity suggest that contextual auditory stimulation may co-occur with systematic changes in neural dynamics during visual word recognition in adults with dyslexia. Variations in alpha (α), beta (β), and gamma (γ) band activity highlight the potential utility of EEG-derived measures for characterizing individual differences in attentional and cognitive processing, rather than implying direct enhancement or remediation effects. From an applied perspective, these findings support the use of spectral EEG markers as exploratory tools for investigating neural variability across task contexts.

Importantly, the present results are correlational and do not provide evidence for the efficacy of music- or rhythm-based interventions. Any educational or therapeutic implications should therefore be considered preliminary. Future research may combine interpretable spectral EEG features with machine-learning approaches, as explored in recent dyslexia detection studies [29], while maintaining transparency regarding underlying physiological mechanisms. Further work should also examine how specific musical parameters (e.g., tempo, structure, familiarity) interact with task demands and individual differences, ideally within controlled and longitudinal designs.

## 5. Conclusions

In conclusion, the present study provides exploratory evidence that neural oscillatory activity during visual word recognition differs between adults with and without dyslexia and varies as a function of contextual and affective factors, including background music and emotional state. Using portable EEG, the findings highlight frequency-band–specific patterns associated with task context at the group level. By integrating oscillatory EEG measures with effective and contextual variables, this work contributes to ongoing efforts to characterize the multifactorial neural dynamics underlying dyslexia. It also underscores the need for future studies employing larger samples, behavioral validation, and longitudinal or interventional designs.

## Figures and Tables

**Table 1:** Independent-samples *t*-test results comparing mean oscillatory power (a.u.) between control and dyslexic participants during visual word recognition without background music. Positive and negative values indicate relative increases or decreases in mean power between groups.

Left Hemisphere						
	Control (n = 14)		Dyslexic (n = 12)		df 25	
Brain Regions_Rhythms	M	SD	M	SD	*t*	Sig
Temporal_Left_*β*	0.142	0.088	0.064	0.060	2.334	0.020
Temporal_Left_*γ*	0.170	0.111	0.080	0.081	2.090	0.040
Occipital_Left_*δ*	0.420	0.132	0.595	0.206	−2.499	0.021
Occipital_Left_*α*	0.045	0.018	0.034	0.011	2.567	0.011
Occipital_Left_*β*	0.106	0.025	0.068	0.039	2.813	0.010
Parietal_Left_*β*	0.140	0.076	0.079	0.048	2.121	0.032
**Right Hemisphere**						
Occipital_Right_*δ*	0.355	0.152	0.538	0.205	−2.612	0.015
Occipital_Right_*β*	0.123	0.041	0.028	0.019	3.136	0.011
Occipital_Right_*γ*	0.131	0.106	0.090	0.062	2.280	0.033

**Note.** Statistical comparisons were performed for all cortical regions (prefrontal, frontal, fronto-central, temporal, parietal, occipital) and all frequency bands (δ, α, β, γ) in both hemispheres. Only region × band combinations yielding a statistically significant between-group difference (*p* < 0.05) are displayed. In the right hemisphere, significant effects under the visual-only condition were confined to the occipital lobe.

**Table 2:** Independent-samples *t*-test results comparing mean oscillatory power (a.u.) between control and dyslexic participants, during visual word recognition with background music. Results indicate between-group differences in spectral power.

Right Hemisphere						
	Control (n = 14)		Dyslexic (n = 12)		df 25	
Brain Regions_Rhythms	M	SD	M	SD	*t*	Sig
Occipital_Right_*γ*	0.019	0.100	0.100	0.100	4.109	**0.050**
Frontal_Right_*β*	0.037	0.028	0.110	0.110	5.709	**0.028**
Frontal_Right_*a*	0.019	0.005	0.054	0.042	10.072	**0.010**
Temporal_Right_*a*	0.029	0.016	0.076	0.062	5.724	**0.038**

**Note.** As in Table 1, statistical comparisons were performed for all cortical regions and frequency bands in both hemispheres. Only region × band combinations yielding a statistically significant between-group difference (*p* < 0.05) are displayed. Under the musical condition, significant effects were confined to right-hemisphere regions. The apparent difference in regional coverage between Table 1 and Table 2 reflects the genuine shift in the distribution of significant effects across conditions, not a change in the scope of the analysis.

**Table 3:** MANOVA results examining group-related differences in oscillatory power (a.u.) across Anxiety tertile categories (DASS-21) during the visual-only condition (without background music). Only statistically significant between-group comparisons are displayed.

	ANXIETY							
	Low	Med	High	Low	Med	High		
	Control (n = 7)			Dyslexic (n = 7)				
Left Hemisphere	M	M	M	M	M	M	*t*	Sig
Frontal_Left_*θ*	0.625			0.510			2.252	0.049
Frontal_Left_*δ*	0.135			0.179			−3.496	0.010
Frontal_Left_*α*	0.029			0.048			−2.952	0.021
Parietal_Left_*α*	0.027			0.051			−2.280	0.047
Occipital_Left_*δ*	0.127			0.162			−2.568	0.037
Occipital_Left_*γ*		0.195			0.048		2.760	0.028
Temporal_Left_*δ*	0.063			0.111			−2.467	0.043
**Right Hemisphere**								
Parietal_Right_*α*	0.044			0.054			−3.786	0.003
Occipital_Right_*δ*	0.123			0.154			−2.941	0.013
Occipital_Right_*γ*		0.204			0.010		2.185	0.050
Temporal_Right_*δ*	0.080			0.134			−2.261	0.045

**Note.** Only region × frequency-band combinations yielding a statistically significant between-group difference (*p* < 0.05) are displayed. Empty cells indicate the absence of a statistically significant effect for that region × band combination and emotional-state tertile; all combinations were tested. The full sample (N = 26) was divided into tertiles based on DASS-21 subscale scores, yielding three emotional-state categories (Low, Medium, High). Due to the limited overall sample size, between-group comparisons within each tertile are based on small subgroups and should be interpreted as exploratory.

**Table 4:** MANOVA results examining group-related differences in oscillatory power (a.u.) across Stress tertile categories (DASS-21) during the visual-only condition (without background music). Only statistically significant between-group comparisons are displayed.

	STRESS							
	Low	Med	High	Low	Med	High		
	Control (n = 7)			Dyslexic (n = 7)				
Right Hemisphere	M	M	M	M	M	M	*t*	Sig
Frontal_Right_*θ*		0.592			0.678		−2.248	0.046
Frontal_Right_*δ*		0.148			0.070		2.744	0.019
Frontal_Right_*α*		0.030			0.016		3.208	0.008
Parietal_Right_*δ*		0.120			0.096		2.171	0.050
Parietal_Right_*α*		0.045			0.024		2.694	0.021

**Note.** Only region × frequency-band combinations yielding a statistically significant between-group difference (*p* < 0.05) are displayed. Empty cells indicate the absence of a statistically significant effect for that region × band combination and emotional-state tertile; all combinations were tested. The full sample (N = 26) was divided into tertiles based on DASS-21 subscale scores, yielding three emotional-state categories (Low, Medium, High). Due to the limited overall sample size, between-group comparisons within each tertile are based on small subgroups and should be interpreted as exploratory.

**Table 5:** MANOVA results examining group-related differences in oscillatory power (a.u.) across Anxiety tertile categories (DASS-21) during the music condition (with background music). Only statistically significant between-group comparisons are displayed.

	ANXIETY							
	Low	Med	High	Low	Med	High		
	Control (n = 7)			Dyslexic (n = 7)				
Left Hemisphere	M	M	M	M	M	M	*t*	Sig
Frontal_Left_*δ*	0.145			0.178			−2.974	0.021
Frontal_Left_*α*	0.030			0.045			−2.480	0.042
Temporal_Left_*α*		0.069			0.021		2.873	0.024
**Right Hemisphere**								
Occipital_Right_*δ*	0.112			0.155			−3.377	0.006

**Note.** Only region × frequency-band combinations yielding a statistically significant between-group difference (*p* < 0.05) are displayed. Empty cells indicate the absence of a statistically significant effect for that region × band combination and emotional-state tertile; all combinations were tested. The full sample (N = 26) was divided into tertiles based on DASS-21 subscale scores, yielding three emotional-state categories (Low, Medium, High). Due to the limited overall sample size, between-group comparisons within each tertile are based on small subgroups and should be interpreted as exploratory.

**Table 6:** MANOVA results examining group-related differences in oscillatory power (a.u.) across Stress tertile categories (DASS-21) during the music condition (with background music). Only statistically significant between-group comparisons are displayed.

	STRESS							
	Low	Med	High	Low	Med	High		
	Control (n = 7)			Dyslexic (n = 7)				
**Right Hemisphere**	**M**	**M**	**M**	**M**	**M**	**M**	***t***	**Sig**
Frontal_Right_*δ*		0.134			0.071		2.262	0.045

**Note.** Only region × frequency-band combinations yielding a statistically significant between-group difference (*p* < 0.05) are displayed. Empty cells indicate the absence of a statistically significant effect for that region × band combination and emotional-state tertile; all combinations were tested. The full sample (N = 26) was divided into tertiles based on DASS-21 subscale scores, yielding three emotional-state categories (Low, Medium, High). Due to the limited overall sample size, between-group comparisons within each tertile are based on small subgroups and should be interpreted as exploratory.

## Data Availability

The raw data supporting the conclusions of this article will be made available by the authors on request.
